# Ultra-High-Performance Liquid Chromatography Coupled with Quadrupole Orbitrap High-Resolution Mass Spectrometry for Multi-Residue Analysis of Mycotoxins and Pesticides in Botanical Nutraceuticals

**DOI:** 10.3390/toxins12020114

**Published:** 2020-02-12

**Authors:** Alfonso Narváez, Yelko Rodríguez-Carrasco, Luigi Castaldo, Luana Izzo, Alberto Ritieni

**Affiliations:** 1 Department of Pharmacy, University of Naples “Federico II”, Via Domenico Montesano 49, 80131 Naples, Italy; alfonso.narvaezsimon@unina.it (A.N.); luigi.castaldo2@unina.it (L.C.); luana.izzo@unina.it (L.I.); ritialb@unina.it (A.R.); 2 Laboratory of Food Chemistry and Toxicology, Faculty of Pharmacy, University of Valencia, Av. Vicent Andrés Estellés s/n, Burjassot, 46100 València, Spain; 3 Department of Clinical Medicine and Surgery, University of Naples "Federico II", Via S. Pansini 5, 80131 Naples, Italy

**Keywords:** mycotoxins, pesticides, Q-Exactive Orbitrap, CBD capsule, nutraceutical

## Abstract

Cannabidiol (CBD) food supplements made of *Cannabis sativa* L. extracts have quickly become popular products due to their health-promoting effects. However, potential contaminants, such as mycotoxins and pesticides, can be coextracted during the manufacturing process and placed into the final product. Accordingly, a novel methodology using ultra-high-performance liquid chromatography coupled with quadrupole Orbitrap high-resolution mass spectrometry (UHPLC-Q-Orbitrap HRMS) was developed to quantify 16 mycotoxins produced by major *C. sativa* fungi, followed by a post-target screening of 283 pesticides based on a comprehensive spectral library. The validated procedure was applied to ten CBD-based products. Up to six different *Fusarium* mycotoxins were found in seven samples, the most prevalent being zearalenone (60%) and enniatin B1 (30%), both found at a maximum level of 11.6 ng/g. Co-occurrence was observed in four samples, including one with enniatin B1, enniatin A and enniatin A1. On the other hand, 46 different pesticides were detected after retrospective analysis. Ethoxyquin (50%), piperonyl butoxide (40%), simazine (30%) and cyanazine (30%) were the major residues found. These results highlight the necessity of monitoring contaminants in food supplements in order to ensure a safe consumption, even more considering the increase trend in their use. Furthermore, the developed procedure is proposed as a powerful analytical tool to evaluate the potential mycotoxin profile of these particular products.

## 1. Introduction

Nutrition is known to be an essential component of the health state, so having an unbalanced diet can lead to several disorders and diseases [1]. Due to current lifestyles, new and fast ways to maintain proper dietary habits are required. Nutraceuticals have emerged as an alternative to increase the input of nutrients, contributing to an improvement in health. These products are bioactive compounds naturally occurring in food or produced de novo in human metabolism, biologicals or botanicals, each intended to impart a physiological or medicinal effect after ingestion [2]. They can be delivered either in foods and beverages or in other non-conventional forms, such as capsules, tablets, powders or liquid extracts. In terms of marketing, nutraceuticals include a large number of different products packaged for specific groups by age, gender, physical conditions and activity level. The global market was valued at US$109 billion in 2015 and is projected to reach US$180 billion by 2020 [3].

Inside the variety of products classified as nutraceuticals, food supplements based on botanical ingredients represent the second largest segment, behind vitamins and minerals. Most recently, cannabidiol (CBD) dietary supplements made of *Cannabis sativa* L. extracts have quickly become popular products. CBD is a phytocannabinoid present in the resin secreted from trichomes in female *C. sativa* plants, and is mainly found in inflorescences. The bioactivity of this compound has been related to an enhancement of its antioxidant and neurological activity, among others, by the promotion of several metabolic pathways [4,5,6]. However, the European Union (EU) does not consider CBD supplements as a novel food [7] and lets member states set their own rules over its marketing, leading to a convoluted situation in terms of regulation. Despite several ambiguities in its legislation, the European market for CBD-based supplements was valued at US$318 million in 2018 and with a strong growth projection [8].

Due to the complex nature of *C. sativa* and other botanicals, potential contaminants can be coextracted during the different stages of the manufacturing process and placed into the final product. Among all the potential non-desirable compounds in herbal-based supplements, mycotoxins and pesticides are the most commonly reported [9,10]. Mycotoxins are secondary metabolites mainly produced by the fungi genera *Fusarium*, *Aspergillus*, *Penicillium*, *Claviceps* and *Alternaria*. These compounds can be present in food and feed commodities and display immunosuppressive, nephrotoxic or carcinogenic effects, among others [11]. According to their carcinogenic potential, some mycotoxins, like aflatoxins, have been included in the classification list of human carcinogens provided by the International Agency for Research on Cancer (IARC) [12]. These mycotoxins are produced by the genera *Aspergillus*, which has been categorized as a major fungus occurring in *C. sativa* inflorescences alongside other mycotoxin producing fungi, like *Fusarium* spp., so different mycotoxins could be also expected [13,14]. On the other hand, pesticides include a broad range of compounds routinely applied to protect crops from different pests. However, residues coming from these products can accumulate in plants intended for human consumption, leading to several health issues related to neurotoxicity, carcinogenicity and pulmonotoxicity, as well as developmental and reproductive disorders [15,16,17,18].

In terms of regulation, maximum residue limits (MRL) for different types of contaminants have been set by the EU. Regulation (EC) No. 396/2005 [19] establishes limits for pesticides, whereas Regulation (EC) No. 1881/2006 [20] covers mycotoxins, attaching maximum limits in food and feeds. Nevertheless, nutraceutical products are not considered by the legislation yet but, due to a potential carryover during the manufacturing process, contamination could be expected not only in raw material, but also in other by-products. Moreover, several studies have reported the sole presence of pesticides [21,22], mycotoxins [23,24] and both types of contaminants [25,26,27] in diverse food supplements, remarking the necessity to evaluate the contamination profile of these products considering their rising consumption and popularity.

To overcome this point, the development of analytical procedures is needed. Concerning the extraction of contaminants, QuEChERS (quick, easy, cheap, effective, rugged and safe) [21,23,24] and “dilute and shoot” procedures have been recently applied to food supplements delivered as gelatin capsules, traditional capsules, tablets, powder extracts or liquid presentations [25,26,27]. Analytical methods used in the detection and quantification of contamination include ELISA detection [28], gas chromatography (GC) coupled with mass spectrometry (MS) [22] and ultra-high-performance liquid chromatography (UHPLC) coupled with tandem mass spectrometry (MS/MS) [23,24] and high-resolution Orbitrap mass spectrometry (Q-Orbitrap HRMS) [25,26,27]. Due to its high resolving power, sensitivity and accurate mass measurement, high-resolution mass spectrometry stands as a suitable alternative for evaluating a large number of contaminants present in complex matrices at low concentrations. Therefore, the aim of the present study was to provide an analysis of pesticide residues and mycotoxins produced by major *C. sativa* fungi occurring in CBD-based food supplements, using ultra-high-performance liquid chromatography coupled with high-resolution Orbitrap mass spectrometry. To achieve this, a novel methodology was developed in order to identify and quantify 16 mycotoxins after evaluating different extraction procedures, followed by a post-target screening of 283 pesticides based on a comprehensive spectral library. To the best of the authors’ knowledge, this is the first multi-class analysis of CBD-based supplements through the use of high-resolution mass spectrometry techniques.

## 2. Results

### 2.1. Optimization of Extraction Procedure

The molecular complexity of this matrix demands an effective extraction in order to detect and quantify several mycotoxins in a reliable way. A QuEChERS methodology previously developed on this typology of sample [24] was selected as the starting point, whereas different volumes of extraction solvent and the type of sorbent for clean-up was tested.

#### 2.1.1. Evaluation of the Volume of Extraction Solvent

The extraction procedure was first evaluated in triplicate by spiking the sample at 10 ng/g using the following volumes of extraction solvent per gram of sample: 2.5, 5, 7.5 and 10 mL.

**Figure 1:**
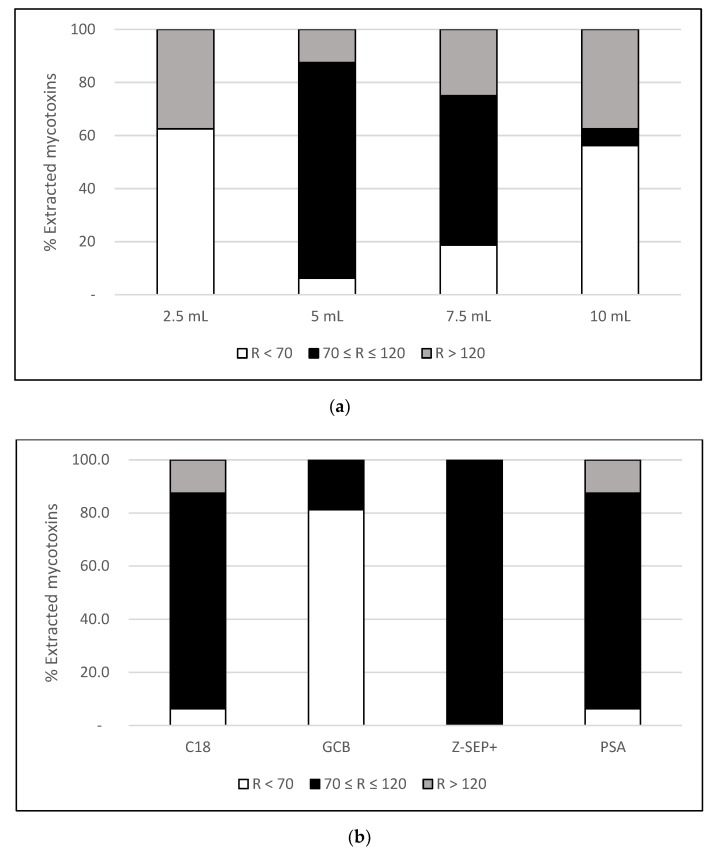
Percentage of mycotoxins extracted with a recovery value (R) below 70% (white), between 70% and 120% (black) and above 120% (grey), corresponding to extractions performed with: (**a**) different volumes of solvent at a spiking level of 10 ng/g; (b) different sorbents for clean-up at a spiking level of 10 ng/g.

The extraction performed with 2.5 mL showed recovery values below the minimum limit (70%) for the vast majority of the studied analytes as a consequence of solvent saturation (Figure 1a). Satisfactory recoveries (70–120%) were obtained after performing the extraction with 5 mL of solvent for the majority of compounds, with the exception of β-ZEL (155%) and ZAN (150%), which were significantly more efficient than the other volumes tested (*p* < 0.05). On the other hand, the extractions performed with 7.5 and 10 mL showed a gradual decrease in recoveries due to the larger dilution of the analytes. Therefore, 5 mL of AcN was selected as the optimal volume of extraction solvent for this type of CBD capsule.

#### 2.1.2. Evaluation of the Type of Sorbent for Clean-Up

The molecular composition of the soft gel capsules mainly consists on fatty acids and proteins. Because of the complex nature of this matrix, an efficient clean-up is required in order to avoid interference with the analytes. To achieve this, clean-up with different sorbents (100 mg), including C18, as previously suggested [24], GCB, Z-Sep+ and PSA was performed.

PSA exhibited a good performance for the vast majority of analytes (Figure 1b) but was unable to recover other important mycotoxins, such as AFB1 and AFG1. The moderate affinity of PSA with polar compounds may explain low recoveries for aflatoxins, being consistent with other works based on oily matrices [29,30]. Similarly, extraction with C18 was efficient for most compounds and only some low-polarity mycotoxins showed recoveries out of the range set, like ZAN (150%) and β-ZEL (155%). Clean-up using GCB showed poor results, allowing us to detect only NEO (85%), HT-2 (89%) and T-2 (89%). This sorbent is able to retain planar molecules and mycotoxin adsorption has been previously reported [31], which might be the reason for the low recoveries obtained here. Finally, extraction performed with Z-Sep+ showed satisfactory recoveries (70–120%) for all the mycotoxins studied.

On the other hand, the influence of the matrix was minimal (80% ≤ signal suppression/enhancement (SSE) ≤ 120%) for all targeted analytes when using Z-Sep+ and PSA. Clean-up based on Z-Sep+ has been successfully applied to the extraction of analytes from lipid matrices [32,33]. Furthermore, Z-Sep+ is also able to form irreversible links with carboxylic groups present in proteins [34], standing as the most suitable sorbent for the here-analyzed matrix. Similarly, the use of PSA has been suggested to remove coextracted fatty acids and other ionic lipids [35].

On the contrary, a strong matrix effect was evidenced for half the analytes when using C18 and GCB. Signal suppression was detected after using C18, obtaining SSE ranging from 40% to 69%, whereas signal enhancement occurred after GCB clean-up, with SSE increasing from 128% to 167%. Since both sorbents have a preferential affinity for non-polar compounds, matrix interferents were not fully removed but coextracted. The presence of these coextracted species can change the ionization efficiency, leading to improper SSE and preventing a reliable quantification. Although no significant differences were observed between the use of Z-Sep+ and PSA (*p* > 0.05), Z-Sep+ was chosen because of its better performance minimizing matrix interference.

### 2.2. Analytical Method Validation

The optimized method was validated for the simultaneous extraction of 16 mycotoxins in CBD-based products. Results are shown in Table 1. Good linearity was observed for all analytes in the range assessed (0.20–100 ng/g), with regression coefficients (r^2^) above 0.990 and a deviation ≤20% for each level of the calibration curve. Comparison between calibration curves built in a blank matrix and in neat solvent showed a minimal interference in the matrix (±20%) for the studied analytes. Hence, external calibration curves were used for quantification purposes. Limits of quality (LOQs) obtained for all studied analytes were between 0.20 and 6.25 ng/g. Regarding trueness, recovery values corresponding to a fortification level of 20 ng/g ranged between 63 and 103% and between 63 and 113% for the lowest fortification level (10 ng/g). Referring to the additional spiking level (2 ng/g) for aflatoxins, recoveries ranged between 63% and 86%. Precision study revealed both RSD_r_ and RSD_R_ values below 20% for all the mycotoxins analyzed. These results confirmed that the optimized procedure is suitable for a reliable quantification of the mycotoxins analyzed, fulfilling the criteria set by Commission Decision 2002/657/EC [36]. Table 2 reviews the available literature regarding mycotoxins in herbal-based supplements. As shown, the here-obtained LOQs were lower than the ones reported in previous studies using UHPLC-Q-Orbitrap HRMS. As established by Regulation (EC) No. 1881/2006 [20], maximum limits for aflatoxins in many food matrices must not reach levels which are below those LOQs (5 ng/g), whereas LOQs obtained in this study were between 5 and 25 times lower. Other analytical methods based on low resolution mass spectrometry [37] required longer and more complicated extraction procedures than the QuEChERS developed here. Even ELISA detection has been used for quantification of mycotoxins in medicinal herbs [28], but a very specific extraction had to be performed for different groups of analytes using several multi-functional columns. The QuEChERS procedure developed in this study, in combination with UHPLC-Q-Orbitrap mass spectrometry, was extremely simple and reliable, allowing for the quantification of all mycotoxins with high sensitivity.

### 2.3. Application to Commercial CBD-Based Products

The validated UHPLC-Q-Orbitrap HRMS procedure was applied to ten commercially available samples in order to evaluate the occurrence of mycotoxins. Results are shown in Table 3. A considerable occurrence of mycotoxins was observed, since contamination with at least one analyte was found in 70% of the samples. Up to six different mycotoxins (T-2, ZAN, ZEN, ENNB1, ENNA, ENNA1) were quantified at a range from below LOQ to 11.6 ng/g, all produced by *Fusarium* genera, reported as a major *C. sativa* pathogen fungus [14]. Previous studies regarding mycotoxins in different herbal-based extracts have revealed the occurrence of similar mycotoxins independently of the matrix and the dosage form (Table 2). Despite the fact that the percentage of positive samples varied among the different studies (19–99%), when the sensitivity of the analytical method increased, reaching lower LOQs, the number of positive samples dramatically increased. This indicated that mycotoxin contamination in herbal-based products at low levels is frequent.

In the here-analyzed samples, ZEN appeared to be the most common mycotoxin, with an incidence of 60% and concentration levels ranging from 4.2 to 11.6 ng/g (mean level = 6.9 ng/g). A high incidence of ZEN has also been previously reported in supplements made of different herbals from Czech and US retail markets (84%, n = 69) at a wide range of concentrations (5–824 ng/g, mean value = 75.7 ng/g) [24]. Moreover, ZEN was previously found in 96% of medicinal herbals from Spain (n = 84) as well, but in a tighter range (1–44.1 ng/g, mean value = 8.9 ng/g) [28].

Referring to T-2, results reported contamination in one sample at 2.0 ng/g, in contrast with the prevalent presence of T-2 in 78% (n = 69) of the same Czech and US samples, at concentrations rising from 69 to 1,870 ng/g (mean value = 162 ng/g) [24]. High levels of T-2 were also observed in milk thistle samples from Spain (363–453.9, mean value = 408.9 ng/g) in only two out of seven samples [38]. In the other hand, T-2 was quantified in 98% (n = 84) of the Spanish medicinal herbals, but in much lower concentrations (0.6–256 ng/g, mean value = 22.645 ng/g) [28].

Similarly, ZAN was quantified in one sample at 1.9 ng/g. This mycotoxin has been scarcely targeted in dietary supplement studies, but has been previously quantified at similar concentrations as those here-reported in two samples of Chinese medicinal herbals (n = 33) [39].

Results also showed ENN contamination. ENNB1, ENNA and ENNA1 were found in the same sample at 11.6, 4.2 and 5.8 ng/g, respectively, whereas ENNB1 was detected in two other samples below the LOQ (1.56 ng/g). These emerging *Fusarium* mycotoxins have been previously found in herbal products (84–91%, n = 69) widely ranging from 5 ng/g up to 10,900 ng/g (mean value = 354 ng/g) [24]. Similarly, ENNB1 was the most common toxin out of these emerging *Fusarium* mycotoxins, being consistent with the results here obtained.

All the mycotoxins found in the present study correspond to low- to non-polar compounds, which should be prevalently expected due to the nature of the matrix.

Co-occurrence of at least two mycotoxins was also observed in four out of ten samples. Results showed the presence of ZEN in combination with ENNs B1, A and A1, ZAN or T-2, which are common associations found by previous studies in herbal-based supplements [24,28]. It must be highlighted that synergic or additive effects have been observed as a consequence of these combinations in in vitro assays [40]. Based on what has been discussed and considering the uprising trend of *C. sativa*-based products, alongside the use of environment-friendly raw materials cultivated without pesticides, quality controls regarding mycotoxins should be set for these products in order to ensure safe consumption.

### 2.4. Identification of Non-Target Compounds through Retrospective Analysis in Studied Samples

**Figure 2:**
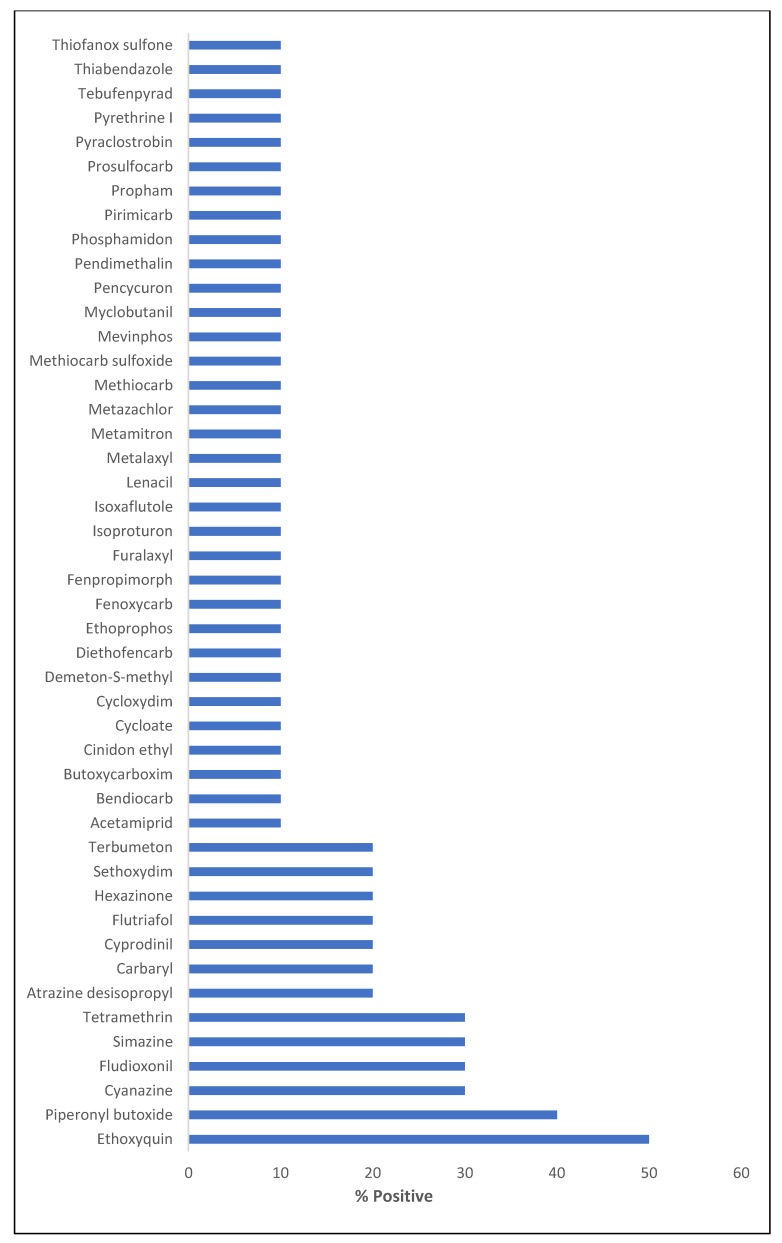
Occurrence of non-target pesticides in analyzed samples after post-run retrospective screening.

The post-target screening approach allowed us to detect pesticide residues in the analyzed samples using a spectral library. Results are shown in Figure 2. Up to 46 different pesticides were tentatively identified based on the pesticides mass spectral library. Ethoxyquin was putatively found in five samples, being the most prevalent pesticide. The main function of ethoxyquin is to avoid fungal contamination during the postharvest stage of the plant through its scald-preventive properties [42]. Surprisingly, the use of this pesticide is forbidden by the European Commission (EC) Decision 2011/143/EU. Piperonyl butoxide was found in four samples. This compound is not a pesticide by itself but can inhibit the resistance mechanisms of insects, being widely used in combination with other different pesticides [42]. The tentative presence of cyanazine and simazine, both found in three different samples, must also be noted. The use of these pesticides was prohibited by EC Regulation No. 1107/2009 [43] and Commission Decision 2004/247/EC [44], respectively. Therefore, the occurrence of forbidden pesticides found in the here-analyzed samples highlights the necessity of monitoring potential contaminants in *C. sativa*-derived products acquired from online shops.

## 3. Conclusions

A sample preparation procedure based on a QuEChERS followed by UHPLC coupled with high-resolution Q-Orbitrap mass spectrometry was optimized in order to determine and quantify 16 mycotoxins in *C. sativa*-based supplements. The proposed methodology was validated following the EU criteria, ensuring a proper specificity, selectivity, linearity, trueness and precision with a fast chromatography run performance (8 min). The validated procedure was applied to ten CBD-based supplements that are commercially available online, allowing us to quantify up to six different *Fusarium* mycotoxins in 70% of samples. ZEN was the most prevalent mycotoxin (60%) found at a maximum level of 11.6 ng/g (mean value = 6.9 ng/g). Co-occurrence was observed in four out of ten samples, including one sample with ENNB1, ENNA and ENNA1. Additionally, a retrospective analysis of pesticide residues was performed. Up to 46 different pesticides were tentatively detected, including some forbidden in *C. sativa* cultivation. Considering the uprising trend of CBD-based products, quality controls regarding contaminants should be set for these products in order to ensure a safe consumption. Furthermore, the developed procedure is proposed as a powerful analytical tool to evaluate the potential mycotoxin profile of these particular products.

## 4. Materials and Methods

### 4.1. Chemicals and Reagents

Acetonitrile (AcN), methanol (MeOH), and water for LC mobile phase (HPLC grade) were acquired from Merck (Darmstadt, Germany). Formic acid and ammonium formate were obtained from Fluka (Milan, Italy). Sodium chloride (NaCl), magnesium sulfate (MgSO_4_), octadecyl carbon chain-bonded silica (C18), graphitized carbon black (GCB), primary-secondary amine (PSA) and zirconium oxide (Z-Sep^+^) were obtained from Sigma Aldrich (Milan, Italy).

Mycotoxin standards and metabolites, namely aflatoxins (AFB1, AFB2, AFG1, and AFG2), HT-2 toxin (HT-2), T-2 toxin (T-2), neosolaniol (NEO), zearalenone (ZEN), α-zearalenol (α-ZEL), β-zearalenol (β-ZEL), zearalanone (ZAN), beauvericin (BEA) and enniatins (ENNA, ENNA1, ENNB, and ENNB1) were purchased from Sigma Aldrich (Milan, Italy). Individual stock solutions of all analytes were prepared by diluting 1 mg of each mycotoxin in 1 mL of methanol. The working standard solution including all the mycotoxins was made by adequate diluting in MeOH:H_2_O (70:30 *v/v*) 0.1% formic acid to reach the required concentrations for performing the spike experiments: 20, 10 and 2 µg/mL. All solutions were kept in safe conditions at −20 °C.

### 4.2. Sampling

For the analysis of real samples, ten different CBD gelatin capsules were obtained from online shops based in different European countries. The capsules are made of gel mass, which contains gelatin, water, glycerin and other minor additives whereas the fill formulation consists of olive oil mixed with hemp oil containing CBD at certain concentrations. The weight of each capsule depended on the manufacturer; there were 0.25, 0.5 and 1 g capsules. Only soft gel capsules were studied since it was the prevalent presentation available for CBD supplements. On the other hand, one sample of CBD supplements delivered as soft gel capsules was acquired from a local store (Naples, Italy). After confirming the absence of contaminants, they were used for preparing fortified samples for recovery assays and matrix-matched standards for calibration purposes. All the samples were conserved in dark and cool conditions, as recommended by the manufacturer, until further analysis.

### 4.3. Sample Preparation

The sample preparation procedure developed by Veprikova et al. [24] was selected as a starting point and then slightly modified, as follows: 1 g of sample was weighed into a 50 mL polytetrafluorethylene (PTFE) tube and mixed with 5 mL of 1% aqueous formic acid. The mixture was placed in an SKO-D XL orbital shaker (Argo Lab, Italy) for 30 min at 294 × *g*. Then, 5 mL of AcN were added and the mixture was shaken for an additional 30 min at 294 × *g*. After that, 0.5 g of sodium chloride and 2 g of magnesium sulfate were added and the tube was shaken for 1 min by hand, followed by centrifugation at 4907 × *g* for 15 min in an SL 16R centrifuge (Thermo Fisher Scientific LED GmbH, Germany). A 2 mL aliquot of the upper acetonitrile layer was taken for dispersive solid phase extraction (d-SPE) cleanup in a 15 mL PTFE tube containing 100 mg of Z-Sep+ sorbent and 300 mg of magnesium sulfate. The tube was vortexed for 1 min and then centrifuged at 4907 × *g* for 15 min. An aliquot of the supernatant (1 mL) was collected and filtered through a 0.2 µm PTFE filter (Phenomenex, Italy) into a vial prior to UHPLC-Q-Orbitrap HRMS analysis.

### 4.4. UHPLC-Q-Orbitrap HRMS Analysis

The qualitative and quantitative profiles of the mycotoxins were obtained using an ultra-high-pressure liquid chromatograph (UHPLC, Thermo Fisher Scientific, Waltham, MA, USA) equipped with a degassing system, a Dionex Ultimate 3000, a Quaternary UHPLC pump working at 1250 bar, an auto sampler device and a thermostated (T = 30 °C) Luna Omega 1.6 µm (50 × 2.1 µm) column.

The eluent consisted of two different phases: A (H_2_O containing 0.1% formic acid and 5 mM ammonium formate) and B (MeOH containing 0.1% formic acid and 5 mM ammonium formate). The gradient elution for LC-Orbitrap HRMS analyses was applied as follows: an initial 0% of phase B was held for 1 min, which linearly went up to 95% B over 1 min and held for 0.5 min. Next, the gradient decreased to 75% B over 2.5 min and then decreased again to 60% B over 1 min. Finally, the gradient turned to 0% B over 0.5 min and then the column was equilibrated for 1.5 min at 0% B. The total run time was 8 min, at a flow rate of 0.4 mL/min. A total of 5 µL of the sample was injected. Detection was performed using a Q-Exactive mass spectrometer. The mass spectrometer was operated in both positive and negative ion mode using fast polarity switching by setting two scan events (full ion MS and all ion fragmentation (AIF)). Full scan data were acquired at a resolving power of 35,000 FWHM at *m/z* 200.

The ion source parameters were: spray voltage 4 kV (-4 kV in ESI− mode); capillary temperature 290 °C; S-lens RF level 50; sheath gas pressure (N_2_ > 95%) 35, auxiliary gas (N_2_ > 95%) 10, and auxiliary gas heater temperature 305 °C. The value for automatic gain control (AGC) target was set at 1 × 10^6^, a scan range of *m/z* 100 to 1000 was selected and the injection time was set to 200 ms. The scan rate was set at 2 scans/s. For the scan event of AIF, the parameters in the positive and negative ion mode were: mass resolving power = 17,500 FWHM; maximum injection time = 200 ms; scan time = 0.10 s; ACG target = 1 × 10^5^; scan range = 100–1000 *m/z*, isolation window to 5.0 *m/z*, and retention time window to 30 s. The Orbitrap-MS parameters were optimized in a previous work [45]. The exact mass for the studied compounds, including elemental composition, retention time (RT), theoretical masses and accurate mass errors for the detected ions are shown in Table 4. A mass error below 5 ppm, referring to the molecular ions, was set for identification. Retrospective screening was carried out on spectral data collected using a pesticide spectral library (Pesticide Spectral Library Version 1.1 for LibraryView™ Software, AB SCIEX, Framingham, USA). For accurate mass measurement, identification and confirmation were performed at a mass tolerance of 5 ppm for the molecular ion and for both fragments at the intensity threshold of 1000. Data analysis and processing were performed using the Xcalibur software, v. 3.1.66.10.

### 4.5. Validation Parameters

An in-house validation study was conducted following the EU Commission Decision 2002/657/EC [36]. The parameters evaluated were selectivity, specificity, linearity, trueness, repeatability (intra-day precision), within-reproducibility (inter-day precision), limit of quantification (LOQ) and limit of detection (LOD). The selectivity and specificity of the method were evaluated by analyzing both standard solutions and samples, comparing the retention time of the peaks corresponding to the analytes of interest alongside the determination of its precursor and product ion, with a mass error below 5 ppm. For linearity, standard solutions built in neat solvent and matrix-matched calibration were analyzed by spiking blank samples at eight concentration levels from 0.2 to 100 ng/g. The slopes of each linear calibration function were compared in order to detect a signal suppression/enhancement (SSE) effect due to the matrix interference. This effect was quantified following the equation: SSE (%) = matrix-matched calibration slope/solvent calibration slope x 100. An SSE value of 100% was interpreted as no matrix interference in the concentration range evaluated. An SSE value above 100% revealed signal enhancement whereas a value below 100% indicated signal suppression. For trueness, recovery studies were evaluated by spiking three blank samples at three different levels. Additionally, a lower spike level was used only for aflatoxins. Intra-day precision (RSD_r_) was expressed as the relative standard deviation after three determinations in a single day (n = 3). Inter-day precision was calculated by repeating the measurements in triplicate on three non-consecutive days (n = 9) and expressed as relative standard deviation (RSD_R_). The LOD was defined as the minimum concentration where the molecular ion can be identified by the instrument (mass error value below 5 ppm) and the LOQ as the minimum concentration where a linear response (mass error value below 5 ppm) can be observed with an accuracy and precision of ≤ 20%.

### 4.6. Statistical Analysis

Validation experiments were performed in triplicate and the results expressed as the average values alongside relative standard deviation (RSD, %). The Saphiro–Wilk test was applied to evaluate normality and multivariant analysis was performed using a non-parametric Kruskal–Wallis test, considering p values < 0.05 as significant. Analysis of data was carried out using IBM SPSS version 25 statistical software package (SPSS, Chicago, IL, USA).

## Figures and Tables

**Table 1:** Method performance: linearity, matrix effect (SSE %), recovery and LOQ.

Analyte	Linearity (r^2^)	SSE (%)	Recovery (%)				Precision (%) [RSD_r_, (RSD_R_)]				
			2 ng/g ^1^	10 ng/g	20 ng/g	50 ng/g	2 ng/g ^1^	10 ng/g	20 ng/g	50 ng/g	LOQ (ng/g)
AFG2	0.9975	111	78	77	81	98	16 (19)	5 (6)	6 (6)	5 (4)	0.78
AFG1	0.9982	106	81	86	86	105	12 (9)	16 (19)	7 (6)	11 (10)	1.56
AFB1	0.9984	115	71	91	98	107	14 (13)	10 (8)	4 (4)	4 (3)	0.20
AFB2	0.9998	111	86	88	91	103	18 (15)	10 (8)	7 (5)	5 (4)	0.20
NEO	0.9988	112		88	93	104		18 (14)	16 (18)	17 (18)	0.78
HT-2	0.9984	108		113	101	92		12 (14)	16 (11)	12 (15)	6.25
T-2	0.9990	83		89	98	110		19 (13)	9 (7)	7 (10)	0.78
α-ZEL	0.9943	81		81	94	100		11 (11)	10 (14)	5 (16)	6.25
β-ZEL	0.9985	84		106	103	89		8 (18)	15 (16)	9 (11)	3.13
ZAN	0.9992	108		111	100	105		15 (13)	18 (11)	5 (13)	1.56
ZEN	0.9991	109		104	103	93		5 (16)	15 (14)	10 (19)	3.13
ENN B	0.9998	102		63	63	65		18 (19)	18 (18)	6 (7)	6.25
ENN B1	0.9982	99		83	89	85		12 (11)	8 (6)	8 (8)	1.56
ENN A	0.9942	84		96	91	80		11 (9)	14 (17)	11 (12)	3.13
ENN A1	0.9972	87		92	101	90		12 (14)	9 (6)	7 (14)	1.56
BEA	0.9971	119		80	71	63		18 (17)	10 (18)	10 (19)	6.25

^1^ Additional fortification level only for AFs.

**Table 2:** Available methods for measurement of mycotoxins in herbal-based supplements ^1^.

Samples Procedence (no.)	Positives Samples (%)	Major Analytes Detected	Concentration Reported (ng/g)	Determination		
				Sensitivity (LOQ, ng/g)	Detection Method	Reference
Medicinal or aromatic herbs (84)	99	ZEN	1.0–44.1	0.14	ELISA detection (EIA reader, SIRIO S)	[28]
		T-2	0.6–256.9	0.28		
		DON^3^	20.5–343.5	14.8		
		CIT^3^	14.9–354.8	16.5		
Traditional Chinese herbs (60)	83	ZEN	2.1–15.5	0.4	QQQ (Applied Biosystems) ESI+ MRM mode	[37]
		AFs^3^	0.2–19.5	0.1		
		MPA^3^	0.2–22.7	0.02		
Milk thistle (83)	19	AFB1	0.04–1.9	0.03	LC-FLD (Waters)	[41]
Green coffee bean (50)	36	OTA^3^	1–136.9	2.5	QQQ (AB SCIEX) ESI^+^ and ESI^-^ MRM mode	[23]
		OTB^3^	1–20.2	2.5		
		FB1^3^	50–415	100		
		MPA	5–395	10		
Milk thistle (7)	29	T-2	363–453.9	30.5	QQQ (AB SCIEX) ESI+ MRM mode	[38]
		HT-2	826.9–943.7	43.8		
Herbals (69)	96	ZEN	5–824	10	QQQ (AB SCIEX) ESI^+^ and ESI^-^ MRM mode	[24]
		T-2	69–1870	10		
		HT-2	59–1530	50		
		ENNB	5–9260	5		
		ENNB1	5–10,900	5		
		ENNA	5–8340	5		
		ENNA1	5–2340	5		
*Gingko biloba* (8)	50	AFB1	5.0–54	5	Q-Orbitrap (Exactive, Thermo FisherScientific) ESI+ and ESI- HRMS	[25]
		AFB2	4–300	10		
		T-2	18–20	30.5		
Green tea (10)	10	AFB1	5.4	5	Q-Orbitrap (Exactive, Thermo FisherScientific) ESI+ and ESI- HRMS	[26]
Royal jelly (8)	0					
Soy (11)	27	AFB1	8.2–17.1	5	Q-Orbitrap (Exactive, Thermo FisherScientific) ESI+ and ESI- HRMS	[27]
		AFG2	6.4	5		
*Cannabis sativa* (10)	70	ZEN	4.2–11.6	3.13	Q-Orbitrap (Exactive, Thermo FisherScientific) ESI+ and ESI- HRMS	Current study
		ENNB1	<LOQ–11.6	1.56		

^1^ ESI+ = positive ion mode; ESI− = negative ion mode; HRMS = high-resolution MS; LOQ = limit of quantification; MRM = multiple reaction monitoring; QQQ = triple quadrupole. ^2^ Range of LOQs referring to the analyzed mycotoxins. ^3^ AFs = aflatoxins; DON = deoxynivalenol; CIT = citrinin; FB1 = fumonisin B1; MPA = mycophenolic acid; OTA = ochratoxin A; OTB = ochratoxin B.

**Table 3:** Occurrence of studied mycotoxins in the analyzed samples.

Sample	Mycotoxin (ng/g)					
	T-2	ZAN	ZEN	ENN B1	ENN A	ENN A1
1			11.6	11.6	4.2	5.8
4			6.5			
5				<LOQ		
7			8.1			
8		1.9	4.7			
9			4.2	<LOQ		
10	2.0		6.3			

**Table 4:** Retention times, accurate mass and mass accuracy of mycotoxins evaluated.

Analyte	Retention Time (min)	Elemental Composition	Adduct Ion	Theoretical Mass (m/z)	Measured Mass (m/z)	Accuracy (Δ ppm)
NEO	4.25	C_19_H_26_O_8_	[M+NH_4_]^+^	400.1966	400.1963	−0.67
AFG2	4.50	C_17_H_14_O_7_	[M+H]^+^	331.0812	331.0808	−1.36
AFG1	4.52	C_17_H_12_O_7_	[M+H]^+^	329.0656	329.0655	−0.27
AFB2	4.58	C_17_H_14_O_6_	[M+H]^+^	315.0863	315.0862	−0.51
AFB1	4.62	C_17_H_12_O_6_	[M+H]^+^	313.0707	313.0705	−0.42
HT-2	4.74	C_22_H_32_O_8_	[M+NH_4_]^+^	442.2435	442.2432	−0.7
α-ZEL	4.83	C_18_H_24_O_5_	[M-H]^-^	319.1551	319.1550	−0.31
T-2	4.85	C_24_H_34_O_9_	[M+NH_4_]^+^	484.2541	484.2543	0.39
β-ZEL	4.97	C_18_H_24_O_5_	[M-H]^-^	319.1551	319.1550	−0.31
ZAN	4.98	C_18_H_24_O_5_	[M-H]^-^	319.1551	319.1549	−0.6
ZEN	5.01	C_18_H_22_O_5_	[M+H]^+^	317.1395	317.1393	−0.54
ENN B	5.56	C_33_H_57_N_3_O_9_	[M+NH_4_]^+^	657.4433	657.4435	0.26
ENN B1	5.68	C_34_H_59_N_3_O_9_	[M+NH_4_]^+^	671.4599	671.4594	−0.76
BEA	5.73	C_45_H_57_N_3_O_9_	[M+NH_4_]^+^	801.4433	801.4432	−0.16
ENN A1	5.82	C_35_H_61_N_3_O_9_	[M+NH_4_]^+^	685.4746	685.4745	−0.18
ENN A	5.99	C_36_H_63_N_3_O_9_	[M+NH_4_]^+^	699.4903	699.4899	−0.56

## References

[1] Afshin A., Sur P.J., Fay K.A., Cornaby L., Ferrara G., Salama J.S., Mullany E.C., Abate K.H., Abbafati C., Abebe Z. (2019). Health effects of dietary risks in 195 countries, 1990–2017: A systematic analysis for the Global Burden of Disease Study 2017. Lancet.

[2] Almada A.L. (2019). Nutraceuticals and functional foods: Innovation, insulation, evangelism, and evidence. Nutraceutical and Functional Food Regulations in the United States and around the World.

[3] Binns C.W., Lee M.K., Lee A.H. (2018). Problems and Prospects: Public Health Regulation of Dietary Supplements. Annu. Rev. Public Health.

[4] Zuardi A. (2008). Cannabidiol: From an inactive cannabinoid to a drug with wide spectrum of action. Rev. Bras. Psiquiatr..

[5] Casares L., García V., Garrido-Rodríguez M., Millán E., Collado J.A., García-Martín A., Peñarando J., Calzado M.A., de la Vega L., Muñoz E. (2019). Cannabidiol induces antioxidant pathways in keratinocytes by targeting BACH1. Redox Biol..

[6] Maroon J., Bost J. (2018). Review of the neurological benefits of phytocannabinoids. J. Surg. Neurol. Int..

[7] (2015). Regulation (EU) 2015/2283 of the European Parliament and of the Council of 25 November 2015 on Novel Foods, Amending Regulation (EU) No 1169/2011 of the European Parliament and of the Council and Repealing Regulation (EC) No 258/97 of the European Parliament and of the Council and Commission Regulation (EC) No 1852/2001.

[8] (2019). International CBD and Cannabis Market Landscape.

[9] Santini A., Cammarata S.M., Capone G., Ianaro A., Tenore G.C., Pani L., Novellino E. (2018). Nutraceuticals: Opening the debate for a regulatory framework. Br. J. Clin. Pharmacol..

[10] Gulati O.P., Ottaway P.B., Jennings S., Coppens P., Gulati N. (2019). Botanical nutraceuticals (food supplements and fortified and functional foods) and novel foods in the EU, with a main focus on legislative controls on safety aspects. Nutraceutical and Functional Food Regulations in the United States and around the World.

[11] Rodríguez-Carrasco Y., Fattore M., Albrizio S., Berrada H., Mañes J. (2015). Occurrence of Fusarium mycotoxins and their dietary intake through beer consumption by the European population. Food Chem..

[12] Ostry V., Malir F., Toman J., Grosse Y. (2017). Mycotoxins as human carcinogens—The IARC Monographs classification. J. Mycotoxin Res..

[13] McKernan K., Spangler J., Zhang L., Tadigotla V., Helbert Y., Foss T., Smith D. (2015). Cannabis microbiome sequencing reveals several mycotoxic fungi native to dispensary grade Cannabis flowers. F1000Res.

[14] McHardy I., Romanelli A., Harris L.J., Opp G., Gaudino R., Torres A., Polage C.R., Tuscano J.M., Thompson G.R. (2018). Infectious risks associated with medicinal Cannabis: Potential implications for immunocompromised patients?. J. Infect..

[15] Atapattu S.N., Johnson K.R.D. (2019). Pesticide analysis in cannabis products. J. Chromatogr. A.

[16] Choudri B.S., Charabi Y. (2019). Pesticides and herbicides. Water Environ. Res..

[17] Mostafalou S., Abdollahi M. (2017). Pesticides: An update of human exposure and toxicity. Arch. Toxicol..

[18] Ye M., Beach J., Martin J.W., Senthilselvan A. (2017). Pesticide exposures and respiratory health in general populations. J. Environ. Sci..

[19] (2005). Regulation (EC) No. 396/2005 of the European Parliament and of the Council of 23 February 2005 on Maximum Residue Levels of Pesticides in or on Food and Feed of Plant and Animal Origin and Amending Council Directive 91/414/EEC.

[20] (2006). Commission Regulation (EC) No. 1881/2006 of the European Parliament and the Council of 19 December 2006 Setting Maximum Levels for Certain Contaminants in Foodstuffs.

[21] Jeong M.L., Zahn M., Trinh T., Brooke F.A., Ma W. (2008). Pesticide residue analysis of a dietary ingredient by gas chromatography/selected-ion monitoring mass spectrometry using neutral alumina solid-phase extraction cleanup. J. AOAC Int..

[22] González-Martín M.I., Revilla I., Betances-Salcedo E.V., Vivar-Quintana A.M. (2018). Pesticide residues and heavy metals in commercially processed propolis. Microchem. J..

[23] Vaclavik L., Vaclavikova M., Begley T., Krynitsky A., Rader J. (2013). Determination of Multiple Mycotoxins in Dietary Supplements Containing Green Coffee Bean Extracts Using Ultra-High Performance Liquid Chromatography-Tandem Mass Spectrometry (UHPLC-MS/MS). J. Agric. Food Chem..

[24] Veprikova Z., Zachariasova M., Dzuman Z., Zachariasova A., Fenclova M., Slavikova P., Vaclavikova M., Mastovska K., Hengst D., Hajslova J. (2015). Mycotoxins in Plant-Based Dietary Supplements: Hidden Health Risk for Consumers. J. Agric. Food Chem..

[25] Martínez-Domínguez G., Romero-González R., Garrido Frenich A. (2015). Determination of toxic substances, pesticides and mycotoxins, in ginkgo biloba nutraceutical products by liquid chromatography Orbitrap-mass spectrometry. Microchem. J..

[26] Martínez-Domínguez G., Romero-González R., Garrido Frenich A. (2016). Multi-class methodology to determine pesticides and mycotoxins in green tea and royal jelly supplements by liquid chromatography coupled with Orbitrap high resolution mass spectrometry. Food Chem..

[27] Martínez-Domínguez G., Romero-González R., Arrebola F.J., Garrido Frenich A. (2016). Multi-class determination of pesticides and mycotoxins in isoflavones supplements obtained from soy by liquid chromatography coupled with Orbitrap high resolution mass spectrometry. Food Control.

[28] Santos L., Marín S., Sanchis V., Ramos A.J. (2009). Screening of mycotoxin multicontamination in medicinal and aromatic herbs sampled in Spain. J. Sci. Food Agric..

[29] Zhao H., Chen X., Shen C., Qu B. (2017). Determination of 16 mycotoxins in vegetable oils using a QuEChERS method combined with high-performance liquid chromatography-tandem mass spectrometry. Food Addit. Contam..

[30] Hidalgo-Ruiz J.L., Romero-González R., Martínez Vidal J.L., Garrido Frenich A. (2019). A rapid method for the determination of mycotoxins in edible vegetable oils by ultra-high performance liquid chromatography-tandem mass spectrometry. Food Chem..

[31] Myresiotis C.K., Testempasis S., Vryzas Z., Karaoglanidis G.S., Papadopoulou-Mourkidou E. (2015). Determination of mycotoxins in pomegranate fruits and juices using a QuEChERS-based method. Food Chem..

[32] Han L., Matarrita J., Sapozhnikova Y., Lehotay S.J. (2016). Evaluation of a recent product to remove lipids and other matrix co-extractives in the analysis of pesticide residues and environmental contaminants in foods. J. Chromatogr. A.

[33] Rajski Ł., Lozano A., Uclés A., Ferrer C., Fernández-Alba A.R. (2013). Determination of pesticide residues in high oil vegetal commodities by using various multi-residue methods and clean-ups followed by liquid chromatography tandem mass spectrometry. J. Chromatogr. A.

[34] Lozano A., Rajski Ł., Uclés S., Belmonte-Valles N., Mezcua M., Fernández-Alba A.R. (2014). Evaluation of zirconium dioxide-based sorbents to decrease the matrix effect in avocado and almond multiresidue pesticide analysis followed by gas chromatography tandem mass spectrometry. Talanta.

[35] Tuzimski T., Szubartowski S. (2019). Method Development for Selected Bisphenols Analysis in Sweetened Condensed Milk from a Can and Breast Milk Samples by HPLC-DAD and HPLC-QqQ-MS: Comparison of Sorbents (Z-SEP, Z-SEP Plus, PSA, C18, Chitin and EMR-Lipid) for Clean-Up of QuEChERS Extract. Molecules.

[36] (2002). Commission Decision 2002/657/EC of 12 August 2002 implementing Council Directive 96/23/EC Concerning the Performance of Analytical Methods and the Interpretation of Results (Text with EEA Relevance).

[37] Han Z., Ren Y., Zhu J., Cai Z., Chen Y., Luan L., Wu Y. (2012). Multianalysis of 35 Mycotoxins in Traditional Chinese Medicines by Ultra-High-Performance Liquid Chromatography–Tandem Mass Spectrometry Coupled with Accelerated Solvent Extraction. J. Agric. Food Chem..

[38] Arroyo-Manzanares N., García-Campaña A.M., Gámiz-Gracia L. (2013). Multiclass mycotoxin analysis in Silybum marianum by ultra high performance liquid chromatography–tandem mass spectrometry using a procedure based on QuEChERS and dispersive liquid–liquid microextraction. J. Chromatogr. A.

[39] Han Z., Ren Y., Zhou H., Luan L., Cai Z., Wu Y. (2011). A rapid method for simultaneous determination of zearalenone, α-zearalenol, β-zearalenol, zearalanone, α-zearalanol and β-zearalanol in traditional Chinese medicines by ultra-high-performance liquid chromatography–tandem mass spectrometry. J. Chromatogr. B.

[40] Smith M.-C., Madec S., Coton E., Hymery N. (2016). Natural Co-Occurrence of Mycotoxins in Foods and Feeds and Their in vitro Combined Toxicological Effects. Toxins.

[41] Tournas V.H., Sapp C., Trucksess M.W. (2012). Occurrence of aflatoxins in milk thistle herbal supplements. Food Addit. Contam..

[42] Pesticides Properties DataBase (PPDB) University of Hertfordshire, United Kingdom. http://sitem.herts.ac.uk/aeru/ppdb/en/.

[43] (2009). Regulation (EC) No. 1107/2009 of the European Parliament and of the Council of 21 October 2009 Concerning the Placing of Plant Protection Products on the Market and Repealing Council Directives 79/117/EEC and 91/414/EEC.

[44] (2004). Commission Decision 2004/247/EC of 10 March 2004 Concerning the Non-Inclusion of Simazine in Annex I to Council Directive 91/414/EEC and the Withdrawal of Authorisations for Plant Protection Products Containing this Active Substance.

[45] Castaldo L., Graziani G., Gaspari A., Izzo L., Tolosa J., Rodríguez-Carrasco Y., Ritieni A. (2019). Target Analysis and Retrospective Screening of Multiple Mycotoxins in Pet Food Using UHPLC-Q-Orbitrap HRMS. Toxins.

